# Effects of intermittent pressure imitating rolling manipulation on calcium ion homeostasis in human skeletal muscle cells

**DOI:** 10.1186/s12906-016-1314-7

**Published:** 2016-08-26

**Authors:** Hong Zhang, Howe Liu, Qing Lin, Guohui Zhang, David C. Mason

**Affiliations:** 1 Yueyang Hospital of Integrative Chinese and Western Medicine Affiliated to Shanghai University of Traditional Chinese Medicine, 100 GanHe Road, Shanghai, 200437 China; 2 University of North Texas Health Science Center, Fort Worth, 76107 USA; 3 Suzhou Health College, Jiangsu, 215009 China

**Keywords:** Rolling manipulation, Human skeletal muscle cell, Calcium ion, Superoxide dismutase, Malondialdehyde, Creatine kinase

## Abstract

**Background:**

Homeostasis imbalance of intracellular Ca^2+^ is one of the key pathophysiological factors in skeletal muscle injuries. Such imbalance can cause significant change in the metabolism of Ca^2+^-related biomarkers in skeletal muscle, such as superoxide dismutase (SOD), malondialdehyde (MDA) and creatine kinase (CK). Measurements of these biomarkers can be used to evaluate the degree of damage to human skeletal muscle cells (HSKMCs) injury. Rolling manipulation is the most popular myofascial release technique in Traditional Chinese Medicine. The mechanism of how this technique works in ameliorating muscle injury is unknown. This study aimed to investigate the possible Ca^2+^ mediated effects of intermittent pressure imitating rolling manipulation (IPIRM) of Traditional Chinese Medicine in the injured HSKMCs.

**Methods:**

The normal HSKMCs was used as control normal group (CNG), while the injured HSKMCs were further divided into five different groups: control injured group (CIG), Rolling manipulation group (RMG), Rolling manipulation-Verapamil group (RMVG), static pressure group (SPG) and static pressure-Verapamil group (SPVG). RMG and RMVG cells were cyclically exposed to 9.5-12.5 N/cm^2^ of IPIRM at a frequency of 1.0 Hz for 10 min. SPG and SPVG were loaded to a continuous pressure of 12.5 N/cm^2^ for 10 min. Verapamil, a calcium antagonist, was added into the culture mediums of both RMVG and SPVG groups to block the influx of calcium ion.

**Result:**

Compared with the CNG (normal cells), SOD activity was remarkably decreased while both MDA content and CK activity were significantly increased in the CIG (injured cells). When the injured cells were treated with the intermittent rolling manipulation pressure (RMG), the SOD activity was significantly increased and MDA content and CK activity were remarkably decreased. These effects were suppressed by adding the calcium antagonist Verapamil into the culture medium in RMVG. On the other hand, exposure to static pressure in SPG and SPVG affected neither the SOD activity nor the MDA content and CK activity in the injured muscle cells regardless of the presence of verapamil or not in the culture medium.

**Conclusion:**

These data suggest that the intermittent rolling pressure with the manipulation could ameliorate HSKMCs injury through a Ca^2+^ dependent pathway. Static pressure did not lead to the same results.

## Background

Chinese medicine massage (also known as Tui Na) has been used over thousands of years as a natural therapy in Chinese clinical settings. Rolling manipulation is a technique in Chinese medicine massage. Its effects on a variety of medical problems, such as muscle pain, have been described in both ancient and modern Chinese medical books [1, 2]. The first book about Chinese Massage (including rolling manipulation) was introduced to western society in 1997 by Maria Mercati [3] and then detailed in many other books written in English including a recent one by Sarah Pritchard [4]. In both books, stimulation of Qi (vital energy) was considered as the mechanism of Chinese massage. The cellular biomechanical effects of Chinese massage, which is presumably more acceptable to western science is still scarcely studied. Our search of literature published in English on this topic only produced two relevant studies. Yi et al. [5] reported that the rolling manipulation could facilitate blood flow at the local area that received the manipulation. Yao et al. [6] found that Chinese massage is able to increase intracellular Ca^2+^ concentration in mast cells of immune system. In general, it is believed that understanding the mechanism of Chinese massage (including rolling manipulation) will help clinicians develop and/or improve the techniques in a more scientifically understandable way [5].

**Fig. 1:**
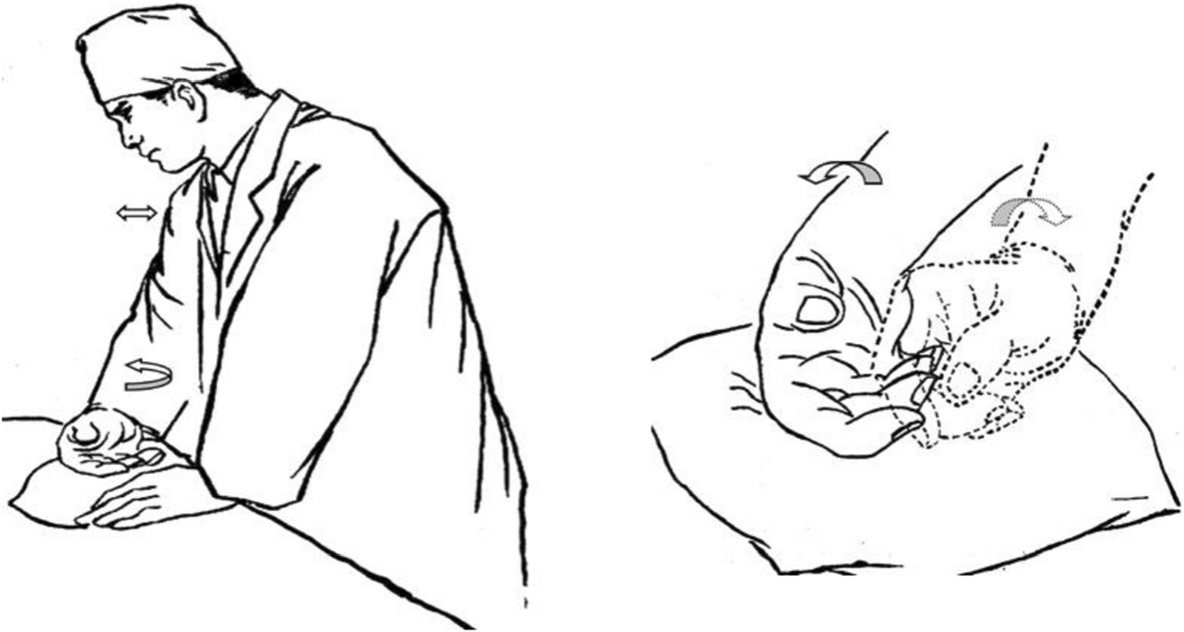
**a** Starting position of rolling manipulation. **b** Hand position changed during operating rolling manipulation

Rolling manipulation is a myofascial release techniques. To perform rolling manipulation in the standing position, the operator is to have his shoulder slightly abducted, elbow and wrist slightly flexed, forearm pronated, hand relaxed in naturally flexed position, with the hypothenar eminence pressing on the body surface of the area being treated on a patient. Performing the technique the operator will have his shoulder slightly more abducted, elbow extending, forearm fully supinating, and wrist slightly extending with their fingers still all naturally flexed. This is a cyclical procedure approximately two cycles per second. During rolling manipulation, the force is transferred to the treated area primarily through elbow extension and forearm supination (Fig. 1). The force produced by the operator’s upper limb will stimulate the body surface in a coordinated rhythmical pattern.

Low back and neck pain are musculoskeletal injuries which manifest as muscle tension, joint stiffness and soft tissue changes with an incidence of more than 50 % in office workers [7]. Rolling manipulation is usually used in the musculoskeletal diseases in clinical settings, and has been confirmed to relieve muscular tension of gastrocnemius muscles by myotonometer [8]. In experimental studies, rolling manipulation on the gastrocnemius muscle is shown to dilate popliteal artery diameter, to decrease vascular resistance, and to increase the average velocity and local tissue blood flow as assessed by Ultrasonic Color Doppler Diagnostic System [9, 10].

Previously, we observed that rolling manipulation with 4.0 kg in maximum pressure, 120 times/min in frequency for 10 min in duration was the optimal condition to improve the increment rate of average volume flow of the popliteal artery [11]. Furthermore, we found that rolling manipulation could activate the secretion function of the human umbilical vein endothelial cells (HUVECs), and promote the synthesis and release of nitric oxide (NO) which is one of endogenous vascular relaxing factors for vasodilatation [12]. We have developed a cell mechanical loading system. In this system, the force curve of rolling manipulation could be imitated and fitted by the biological material test system (MTS, type 858; MTS Company, Eden Prairie, USA). Muscle cells could be loaded by the intermittent pressure imitating the pressure-time curve of rolling manipulation through air pressure [13].

Traditional Chinese Manipulation is effective in treating disease through stimulating the body surface in a rhythmical pattern. The manipulation force could be converted into its biological effects to improve clinical symptoms. However, the mechanism of how the manipulation force could initiate or trigger these biological effects is still unknown.

Homeostasis imbalance of intracellular Ca^2+^ is believed to be one of the key checkpoints during skeletal muscle injuries [14, 15]. Under physiological conditions, Ca^2+^ concentration in the sarcoplasmic reticulum, endoplasmic reticulum and mitochondria of the skeletal muscle cells fluctuates in a small range and maintains at an equilibrium state [16]. Under pathological conditions, Ca^2+^ concentration in the cytoplasm of the skeletal muscle cells increases. Such Ca^2+^ imbalance is associated with the significant changes of the biomarkers of skeletal muscle metabolism such as decreased superoxide dismutase (SOD), increased malondialdehyde (MDA) and increased creatine kinase (CK) in the injury muscle cells [17]. Therefore, changes of these biomarkers in skeletal muscles could be used as an indicator of muscle injury.

To relieve muscle spasm is an important criterion for the evaluation of the therapeutic effect in Chinese manipulation. The mechanical stimulation during the manipulation may further be converted to different biological effects to achieve its therapeutic effect [18]. The key structure responsible for such signal conversion is the mechanical pressure receptor. The potential biochemical structures involved in this process include the ion channels, G-protein, tyrosine kinase and integrin family, among which the ion channels aroused much research interest [19]. It has been reported that the mechanical force from the manipulation could stimulate the ion channels in the cells, which activate the relevant signal pathways [20]. These ion channels include Ca^2+^, Na^+^ and K^+^ channels in the cells, in which Ca^2+^ channel is the focus as detailed below.

The Ca^2+^ channel, which is also named the dihydropyrimidine receptor (DHPR), has dual functions as the voltage sensor and L-type voltage-gated Ca^2+^ channel [21]. The channel could couple the cell membrane depolarization with the Ca^2+^ release from the sarcoplasmic reticulum, also could be the responder of mechanical stimulation [22, 23]. As an intracellular secondary messenger, Ca^2+^ plays an important role in signal transduction, and is necessary for many important enzymes to be activated. Therefore, Ca^2+^ might take part in receiving the mechanical stimulation,in causing the morphological and functional changes in cells, and in converting the mechanical stimulation into the biochemical signals in cells [24, 25].

Hands-on rolling manipulation has been practiced by doctors for centuries to treat their patients with musculoskeletal dysfunctions. However, how the biological mechanism of therapeutic effects is achieved through the mechanical manipulation is still unknown. The aim of this study was to investigate how the manipulation pressure/force could trigger its biological effects with special focus on the possible calcium ion-mediated effects in human skeletal muscle cells.

## Methods

This is a study with cross-sectional design.

### Development of a novel cell mechanical loading system

**Fig. 2:**
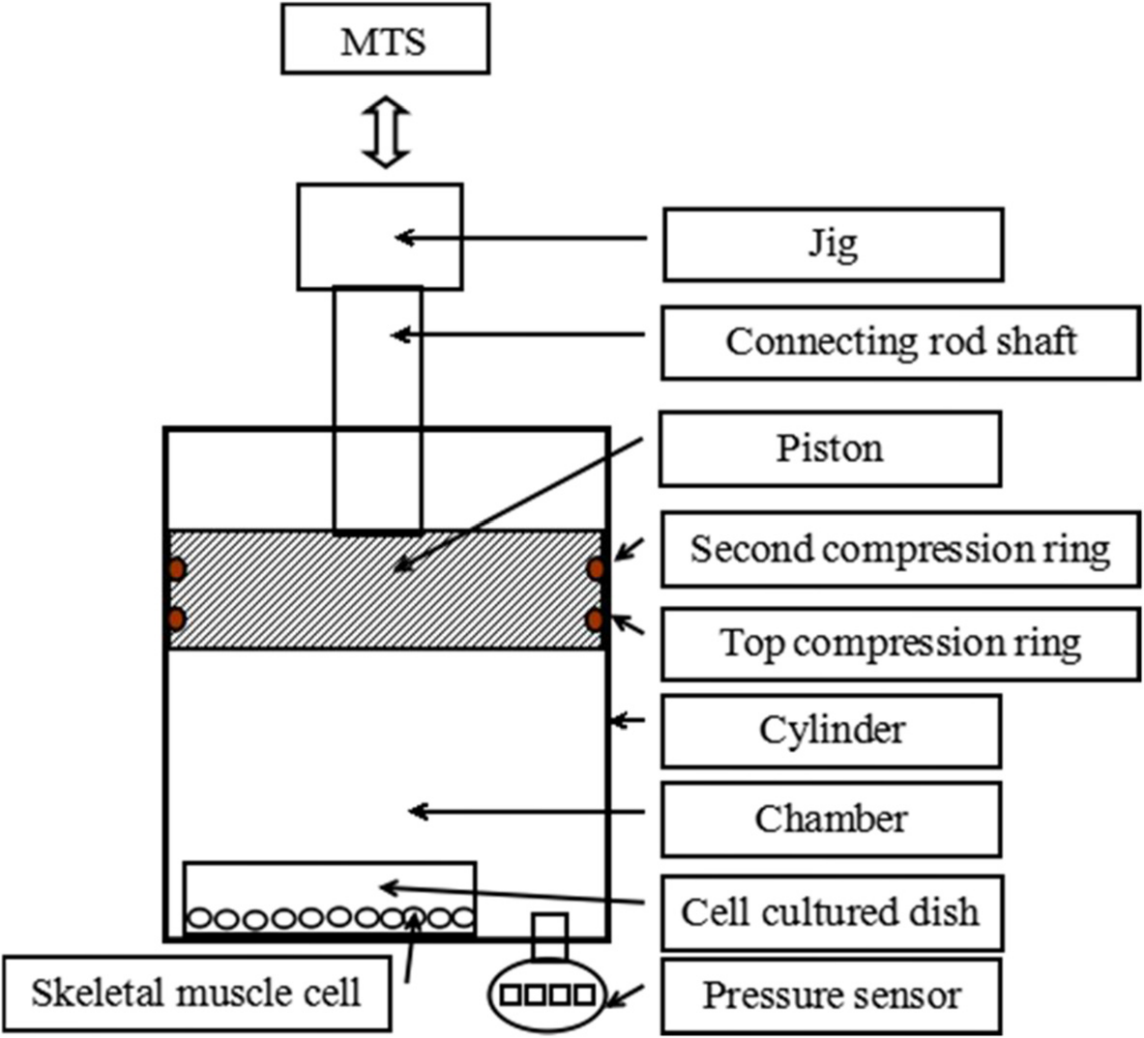
Schematic diagram of the mechanical pressure loading system for living muscle cells

A newly developed mechanical pressure loading system for living muscle cells is shown in Fig. 2. The system consisted of a biological material test system (MTS, type 858; MTS Company, Eden Prairie, USA), a jig, a connecting rod shaft, a piston with two silicone compressing rings for sealing, a stainless steel cylinder as pressure vessel, and a pressure sensor (EVT100A; Yuran Sensor Technology Company, Shanghai, China). The pressure was generated within a stainless steel cylinder interfaced to MTS, a servo-hydraulic loading frame. The cell-culture dish was put on the bottom of the chamber. The chamber was completely closed to create a pressure chamber.

### Curve fitting of rolling manipulation

The pressure-time curve of rolling manipulation was recorded in the Manipulation Technique Parameter Analyzer (TypeII, Shanghai Research Institute of Traditional Chinese Medicine, China) when the operator was performing rolling manipulation. The data of the pressure-time curve were imported into the Waveform Editor in MTS, and MTS output the load to the Cell Mechanical Loading System imitating rolling manipulation.

**Fig. 3:**
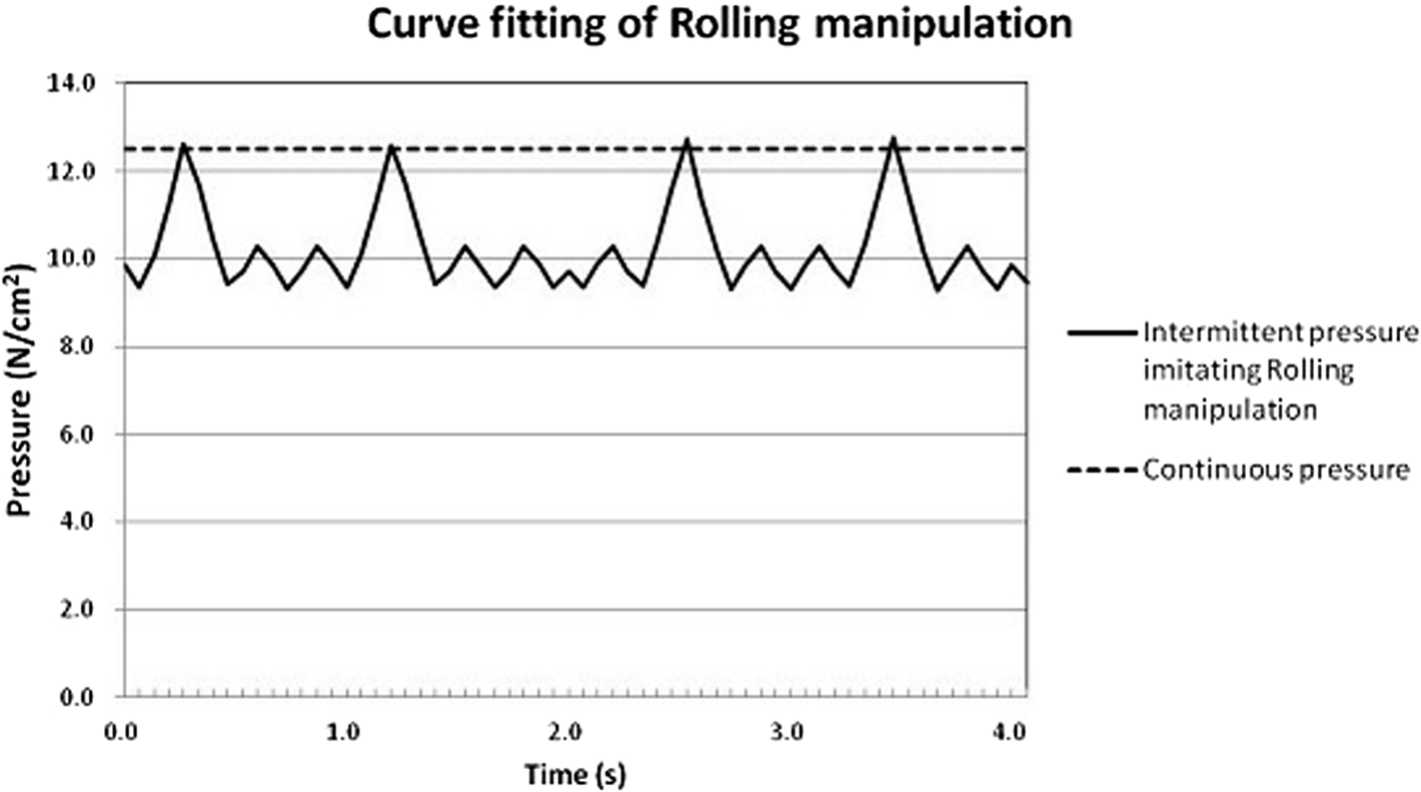
The full curve was the pressure-time curve of IPIRM recorded by the pressure sensor in the Cell Mechanical Loading System. The dotted curve was recorded under a continuous pressure

The full curve in Fig. 3 was monitored by the pressure sensor in the Cell Mechanical Loading System, which showed that the cells were cyclically exposed to the 9.5-12.5 N/cm^2^ of intermittent pressure imitating rolling manipulation (IPIRM) at a frequency of 1.0 Hz. The dotted curve in Fig. 3 showed that the cells were loaded at a continuous pressure of 12.5 N/cm^2^.

### Cell culture and establishment of injured cell model

The human skeletal muscle cells (HSKMCs; US Type Culture collection warehousing, San Diego, USA) from the 4 – 8th generation of skeletal muscle cell strain were used for this study. All cells were kept in CO_2_ cell culture box (Biorad Company, Hercules, USA) at 37 °C in a humidified atmosphere containing 5 % CO_2_. HSKMCs were cultured in DMEM high-glucose medium (HyClone Company, Logan, USA) containing 4.5 g glucose, 100,000U penicillin, 100 mg streptomycin, and 3 % fetal calf serum (FCS; HyClone Company, Logan, USA) per liter. They were considered to be cultured successfully when the following four criteria were identified under an inverted microscope: 1. The shape of human skeletal muscle cells was spindle-shaped. 2. No floating cells were found which indicated that the cultured cells had a good capacity of cellular adherence to wall of the culture bottle. 3. The cell nuclei were oval-shaped without any sign of breaking out, dissolving, or pyknosis. 4. The culture bottles were clear without pollution. Once the HSKMCs grew up to the whole bottom of each culture bottle, they were harvested and divided into two portions in order to re-proliferate further down.

The human skeletal muscle injury modeling cells were induced by dexamethasone according to the reference literature [26]. Some researches conformed that excessive dosage of dexamethasone could produce injury to muscle cells by suppressing cells proliferation, reducing SOD level, increasing MDA, and causing intracellular Ca ^2+^ overloading [27, 28].

When HSKMCs in the culture bottle showed good adherence, i.e., covering 80-90 % on the bottom of the bottle observed under microscope, the cultured cells were harvested and then the medium in the bottle was abandoned. The mixture of the fresh DMEM high-glucose medium and dexamethasone sodium phosphate injection (The 3rd Pharmaceutical Factory, Jiangshu, China) was added into the culture bottle, and the final concentration of Dexamethasone sodium phosphate injection was 2.5 g/L. The cells were then cultured in the incubator containing 5 % CO_2_ for 24 h at 37 °C.

### Groups and treatment of HSKMCs

The normal HSKMCs were used as control normal group (CNG), and they were cultured in 12 dishes. The injured HSKMCs were future divided respectively into 5 following different groups with 12 dishes per group: control injured group (CIG), rolling manipulation group (RMG), rolling manipulation-verapamil group (RMVG), Static pressure group (SPG) and Static pressure-verapamil group (SPVG).

CNG (control normal group) and CIG (control injury group) cells were cultured in the same conditions as RMG, RMVG, SPG and SPVG cells except being loaded pressure. RMG and RMVG cells were cyclically exposed to 9.5-12.5 N/cm^2^ of IPIRM at a frequency of 1.0 Hz for 10 min. SPG and SPVG cells were loaded to a continuous pressure of 12.5 N/cm^2^ for 10 min. In both RMVG and SPVG, verapamil hydrochloride injection (Ver; Wellhope Pharmaceutical Company, Shanghai, China), a calcium ion influx inhibitor, was added into the culture medium with a concentration of 10^-5^ mol/L.

### Procedure of measuring SOD activity, MDA content, and CK activity

The medium were removed by aspiration from culture vessels of each group cells described above. 1 ml of 0.25 % trypsin (Jibco Company, Grand Island, USA) was added into culture vessels, which were placed in 37 °C incubator for approximately 1.5 min. The trypsin was removed by aspiration until HSKMCs appeared rounded when they were observed using an inverted microscope. 6.0 ml of DMEM high-glucose medium were added into culture vessels to terminate trypsinization process. HSKMCs were collected at 1000 r/min for 10 min, and preserved in the refrigerator at -20 °C.

SOD activity, CK activity, and MDA content were quantified in the same experiment and in duplicates with the use of commercially available SOD, CK and MDA kits (Nanjing Jiancheng Bioengineering institute, Jiangsu, China).

### Statistical analysis

All data were continuous data that were expressed as mean ± standard deviation $\left(\overline{x}\pm \mathrm{s}\right)$. One-way Analysis of Variance (ANOVA) with post-hoc multiple comparisons was conducted to analyze the differences between different groups. All the data were analyzed with software (Statistical Package for the Social Sciences, version15.0). A *p*-value of less than 0.05 was considered to be statistically significant.

## Results

### Rolling manipulation pressure-time curve effects on SOD activity of HSKMCs

As shown in Table 1, the SOD activity in the injured HSKMCs in CIG was remarkably decreased as compared with that of the normal HSKMCs in CNG(*P* < 0.05), demonstrating a decreased SOD activity with the muscle cell injury. However, the SOD activity in RMG was significantly higher than that of CIG (*P* < 0.05), indicating that IPIRM could reverse the SOD decrease in the injured HSKMCs. Meanwhile, the SOD activity in RMVG was significantly decreased than that of RMG (*P* < 0.05), showing that the ameliorating effect of intermittent pressure on the SOD could be suppressed by the presence of the Ca^2+^ channel antagonist.

**Table 1:** Comparison of SOD, MDA, and CK in different groups of HSKMCs

Groups	SOD (U/mg prot)	MDA (nmol/mg prot)	CK (U/mg prot)
Numbers of cases	*n* = 10	*n* = 12	*n* = 12
*CNG*	26.06 ± 6.92	1.69 ± 0.36	1.90 ± 0.43
*CIG*	17.63 ± 5.48^*^	2.90 ± 0.51^*^	2.48 ± 0.68^*^
*RMG*	34.70 ± 3.80^**^	2.10 ± 0.72^**^	1.94 ± 0.70^**^
*RMVG*	22.80 ± 5.71^***^	2.49 ± 0.35	2.18 ± 0.68
*SPG*	19.65 ± 3.39^***^	3.09 ± 0.72^***^	2.44 ± 0.61
*SPVG*	17.08 ± 3.36	2.75 ± 0.46	2.55 ± 0.61

*SOD* superoxide dismutase, *MDA* malondialdehyde, *CK* creatinkinase, *CNG* control normal group, *CIG* control injured group, *RMG* rolling manipulation group, *RMVG* rolling manipulation-verapamil group, *SPG* static pressure group, *SPVG* static pressure-verapamil group. What comparing with CNG, *meant *P* < 0.05; What comparing with CIG, ** meant *P* < 0.05; What comparing with RMG, *** meant *P* < 0.05

The SOD activity in SPG was not different from that of GIG and SPVG, but was significantly lower than that of RMG (*P* < 0.05), indicating that the static pressure had no effect on SOD activity in the injured HSKMCs.

### Effect of rolling manipulation on MDA content of HSKMCs

As shown in Table 1, the MDA content in the injured HSKMCs in CIG was significantly increased as compared with that of the normal HSKMCs in CNG (*P* < 0.05), demonstrating an increase of MDA after the muscle cell injury.

The MDA content in RMG was remarkably decreased as compared with that of CIG (P < 0.05), indicating that IPIRM could reduce MDA content in the injured HSKMCs. Meanwhile, the MDA content in RMVG was not different from than that of CIG (*P* > 0.05), which showing that intermittent pressure did not reduce MDA content in the injured HSKMCs with the presence of the calcium antagonist. In other words, the effect of intermittent pressure on MDA content could be suppressed when the mechanical signal was blocked by the Ca^2+^ channel antagonist. The MDA content in the injured HSKMCs in SPG was not different from that of GIG and SPVG, but was significantly higher than that of RMG (*P* < 0.05), indicating that the static pressure had no effect on MDA content in the injured HSKMCs.

### Effect of rolling manipulation on CK activity of HSKMCs

As shown in Table 1, the CK activity in the injured HSKMCs in CIG was obviously increased as compared with that of the normal HSKMCs in CNG (*P* < 0.05), demonstrating a CK release after the muscle cell injury.

The CK activity in RMG was significantly lower than that of CIG (*P* < 0.05), indicating that IPIRM could ameliorate the CK release after the muscle cell injury in HSKMCs. Meanwhile, the CK activity in RMVG was not different from that of CIG (*P* > 0.05), showing that the ameliorating effect of the intermittent pressure on the muscle cell injury could be blocked by the presence of the Ca^2+^ channel antagonist.

Furthermore, the CK activity of the injured HSKMCs in SPG was not different from that in GIG and SPVG, indicating that the static pressure had no effect on the CK activity of injured HSKMCs.

## Discussion

Rolling manipulation in traditional Chinese Medicine may affect human tissue and structures through the mechanical effects. These mechanical effects can be converted into its biological effects to ameliorate the clinical symptoms. The muscle cells are the basic function units in human body, and the final target of the manipulation force. How the muscle cells recognize the rolling changes of the mechanical force, and then convert the signals into some kind of physiological and chemical signals which further causes a series of its biological effects is the key to explain the mechanism of the rolling manipulation.

In our present study, the SOD activity, the MDA content and the CK activity were used as the biomarkers of the cultured muscle cell injury model and further examined after exposure to various experimental conditions.

As compared with that of the normal HSKMCs in CNG, the SOD activity was significantly decreased while both the MDA content and the CK activity were evidently increased in the injured HSKMCs in CIG. With IPIRM, the SOD activity was increased whereas the MDA content and the CK activity were decreased in the injured HSKMCs in RMG as compared with CIG. However, these effects could be further blocked by the presence of the calcium antagonist Verapamil in the culture medium in RMVG. Similar phenomenon were observed in the previous studies where the biological effects of mechanical stimulation on the osteoblasts can partially be inhibited by the L-type calcium channel antagonist nifedipine [29, 30]. On the other hand, the static pressure in SPG and SPVG showed neither effect on SOD activity nor the MDA content and the CK activity in the injured HSKMCs as compared with CIG in our study.

### Effects on SOD

Lipid peroxidation plays an active part in chronic skeletal muscle injuries. The accumulation of oxygen free radicals can cause an increase of the lipid peroxidation, which leads to the damage of the cell structure and functions [31]. Superoxide dismutase (SOD) is an enzymatic defense mechanism in the contingency procedure to defend the lipid peroxidation, and is also one of the major antioxidant enzymes in human body especially in musculature [32].

Our study showed that SOD activity in the injured HSKMCs in CIG was remarkably decreased than that of CNG. With the treatment of intermittent pressure imitating rolling manipulation, the SOD activity was significantly increased in the RMG. This indicated that IPIRM could increase the SOD activity and enhance the antioxidant ability of injured HSKMCs. This result is in agreement with the previous report that the SOD activity was increased by the manipulation intervention like intermittent pressure [33]. Furthermore, the SOD activity was significantly decreased in RMVG with the presence of verapamil in RMVG than that of RMG. In other words, the effect of intermittent pressure on the SOD activity in the injured muscle cells could be blocked by the presence of the calcium channel antagonist. This suggests that the responsive increase of the SOD activity in the injured HSKMCs treated with IPIRM depends on the Ca^2+^ intracellular influx. This result is similar to previous findings that Ca^2+^ concentration in the cytoplasm is negatively related to the activity of SOD [34, 35]. Furthermore, SOD activity in SPG was not different from that of GIG and SPVG, but was significantly lower than that of RMG, indicating that the static pressure had no effect on the SOD activity in the injured HSKMCs.

### Effects on MDA

Cellular Malondialdehyde (MDA) content reflects the active status of oxygen free radicals in the injured tissue. Lipid peroxidation caused by oxygen free radicals is closely related to the skeletal muscle injury [36, 37]. Similar to tissue SOD activity, the MDA content is another biomarker commonly used for chronic skeletal muscle injury. In our study, the MDA content in the injured HSKMCs in CIG was significantly increased as compared with that of the normal HSKMCs in CNG, demonstrating the existence of the muscle cell injury in the CIG. However, the MDA content in the injured HSKMCs in SPG was not different from that of CIG and SPVG, indicating that the static pressure had no effect on ameliorating muscle cell injury in the injured HSKMCs.

With intermittent pressure imitating rolling manipulation (IPIRM), the MDA content in RMG was remarkably decreased in the injured muscle cells as compared with that of CIG, indicating that the ameliorating effect of IPIRM in the injured HSKMCs may be achieved through an improvement of the dynamic balance of lipid peroxidation in the injured HSKMCs. Similar results were reported previously where the MDA content was significantly increased in rat triceps after exhaustive downhill run, which could remarkably be decreased by the delivery of vitamin E, one product against lipid peroxidation caused by the oxygen free radical [38].

The MDA content in RMVG was not different from that of CIG. The fact that the the ameliorating effect of IPIRM in the injured HSKMCs on the MDA content was blocked by the presence of verapamil in the culture medium suggests that this ameliorating process is dependent on the cellular influx of the Ca^2+^ as a messenger.

### Effects on CK

The CK activity in the skeletal muscle cells is closely associated with the transmembrane flow of Ca^2+^ and excitation-contraction coupling [39, 40]. CK in the muscle cells involves mainly in the ATP synthesis. In our study, the CK activity was significantly increased in the injured HSKMCs in CIG as compared with that of the normal HSKMCs in CNG, reflecting the reliability of our muscle cell injury model. With the exposure to the intermittent pressure IPIRM, the CK activity returned to the same level as that of the normal control group in RMG. This gives direct evidence that IPIRM ameliorates the muscle cell injury in our HSKMCs.

Meanwhile, the CK activity in RMVG was not different from that of CIG, showing that the intermittent pressure could not reduce CK activity in the injured HSKMCs with the presence of the calcium channel antagonist verapamil. In other words, the ameliorating effect of intermittent pressure on the muscle cell injury was partially blocked by the Ca^2+^ channel blocker. So it could be inferred that Ca^2+^ might play a triggering role for the biological effects of IPIRM as a so called messenger. Furthermore, the CK activity in the injured HSKMCs in SPG was not different from that of GIG and SPVG, indicating that the static pressure had no ameliorating effect on the muscle cell injury in the injured HSKMCs.

## Conclusions

These results suggest that there is a significant correlation between the calcium ion and the biological effects of the IPIRM in the injured HSKMCs. The Ca^2+^ channel might work as a responder to the mechanical stimulation, and the Ca^2+^ influx might be a key to trigger some kinds of downstream processes to ameliorating the muscle cell injury. It could be assumed that intermittent pressure imitating rolling manipulation could initiate Ca^2+^ channel activation and consequently regulate the influx of Ca^2+^ into the injured HSKMCs, and ultimately improve the functions of these injured muscle cells.

However, there is no significant difference in the biomarkers MDA or CK between the RMG and the RMVG groups. Whether the beneficial effects of rolling manipulation are partially or completely mediated by Ca^2+^ is still unknown based on current results. Therefore, further studies by testing other parameters will be needed in future.

## Abbreviations

BCA: Bicinchoninic acid
Ca^2+^: Calcium ion
CIG: Control injured group
CK: Creatinkinase
CNG: Control normal group
CO_2_: Carbon dioxide
DMEM: Dulbecco’s minimum essential medium
DMSO: Dimethyl sulphoxide
FCS: Fetal calf serum
HSMCs: Human skeletal muscle cells
HUVECs: Human umbilical vein endothelial cells
IPIRM: Intermittent pressure imitating rolling manipulation
MDA: Malondialdehyde
MTS: Mechalical testing simulation
NO: Nitric oxide
NP-40: Nonidet P 40
PBS: Phosphate buffered solution
RMG: Rolling manipulation group
RMVG: Rolling manipulation-verapamil group
rpm: Revolutions per minute
SOD: Superoxide dismutase
SPG: Static pressure group
SPVG: Static pressure-verapamil group
